# The unexpected loss of the ‘hunger hormone’ ghrelin in true passerines: a game changer in migration physiology

**DOI:** 10.1098/rsos.242107

**Published:** 2025-03-19

**Authors:** Stefan Prost, Jean P. Elbers, Julia Slezacek, Alba Hykollari, Silvia Fuselli, Steve Smith, Leonida Fusani

**Affiliations:** ^1^ Ecology and Genetics Research Unit, University of Oulu, Oulu, Finland; ^2^ Interdisciplinary Life Sciences, University of Veterinary Medicine, Vienna, Austria; ^3^ Life Sciences and Biotechnologies, University of Ferrara, Ferrara, Italy; ^4^ Department of Behavioral and Cognitive Biology, University of Vienna, Vienna, Austria

**Keywords:** avian genomics, gene loss, bird migration, comparative genomics

## Abstract

Migratory birds must accumulate large amounts of fat prior to migration to sustain long flights. In passerines, the small body size limits the amount of energy stores that can be transported, and therefore birds undergo cycles of extreme fattening and rapid exhaustion of reserves. Research on these physiological adaptations was rattled by the discovery that birds have lost the main vertebrate regulator of fat deposition, leptin. Recent studies have thus focused on ghrelin, known as ‘hunger hormone’, a peptide secreted by the gastrointestinal tract to regulate, e.g. food intake and body mass in vertebrates. Studies on domestic species showed that, in birds, ghrelin has effects opposite to those described in mammals such as inhibiting instead of promoting food intake. Furthermore, recent studies have shown that ghrelin administration influences migratory behaviour in passerine birds. Using comparative genomics and immunoaffinity chromatography, we show that ghrelin has been lost in Eupasseres after the basic split from Acanthisitti about 50 Ma. We found that the ghrelin receptor is still conserved in passerines. The maintenance of a functional receptor system suggests that in Eupasserines, another ligand has replaced ghrelin, perhaps to bypass the feedback system that would hinder the large pre-migratory accumulation of subcutaneous fat.

## 1. Introduction

Twice a year, billions of birds migrate between breeding and wintering areas—a journey that is very strenuous and often requires stop-over events to replenish fat storage. Migratory birds have the unique ability of storing an enormous amount of subcutaneous fat prior to and during migration (up to 100% of the lean body mass in some species, e.g. [1]). In mammals, the amount of fat stored in the adipose tissue is mainly regulated through a feedback system by the hormone leptin. However, leptin has lost this regulatory function in birds (reviewed in [2]). The disruption of the well-conserved leptin feedback system was suggested to be linked to the need for accumulating large fat storages in a short period of time to sustain long-distance migration [2]. Therefore, recent studies focused on the gastrointestinal hormone ghrelin. Ever since its discovery in 1999 [3], several studies have revealed important roles of this peptide hormone in the central regulation of food intake, body mass, adiposity, glucose metabolism, sleep, anxiety and stress [4–6], among several other functions (see [7] for a review). The activation of ghrelin (gene symbol: GHRL) via acetylation is catalysed by an enzyme called membrane-bound O-acyltransferase domain containing 4 (MBOAT4; [8]). Ghrelin binds as an agonist to the growth hormone secretagogue receptor (GHS-R). There are several forms of GHS-R; however, tetrapods share one single, conserved receptor called GHS-R1a [9]. Liver-enriched antimicrobial peptide 2 (LEAP2) was identified as an antagonist to ghrelin [10].

The functions of ghrelin in birds have been investigated mainly in a few domestic species, where it appears to have opposite effects compared with mammals, i.e. it inhibits food consumption and downregulates the build-up of fat storage [11]. Recent studies showed effects of ghrelin on food intake and migratory behaviour in passerines [12–14]. Ghrelin administration induced an increase in migratory restlessness and a decrease in food intake in garden warblers (*Sylvia borin*; [12]), and an earlier departure from stop-over sites in yellow-rumped warblers (*Setophaga coronata coronata;* [14]), whereas Henderson *et al.* [13] showed that injected ghrelin reduces food hoarding and mass gain in the coal tit (*Periparus ater*). To gain additional knowledge on the involvement of the ghrelin system in migratory behaviour of passerine birds, we conducted comparative genomic analyses. Unexpectedly, we discovered that Eupasserines, that is the ‘true passerine’ taxon that comprises all but two of the more than 6500 passerine species, have lost the gene coding for ghrelin, although they seem to maintain a functional receptor system. We further verified our findings using immunoaffinity chromatography. These surprising results indicate that in addition to leptin, true passerine birds have lost the second main vertebrate regulator of food intake and body mass. This major evolutionary transition might be functional to sustain the need for rapid, repeated gains of large energy stores in small passerines that typically cannot transport all fuel required for the entire migratory journey.

## 2. Results and discussion

### 2.1. Presence and absence of genes upstream of the growth hormone secretagogue receptor in Eupasserine birds

**Figure 1.**
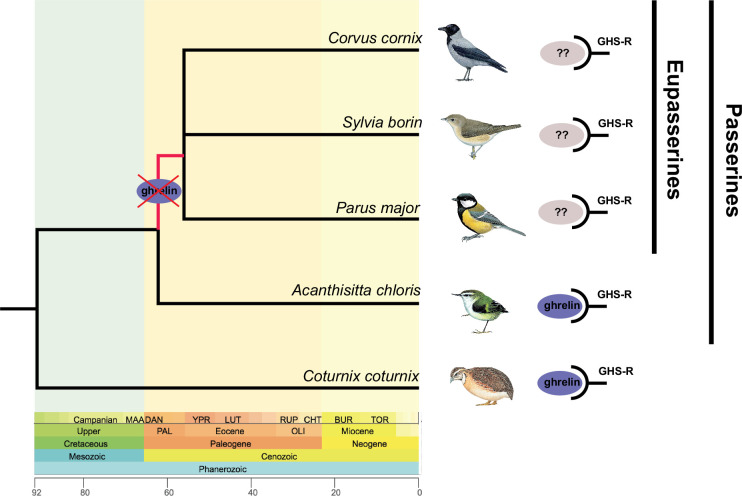
Phylogenetic tree showing the loss of ghrelin in Eupasserine birds. The figure illustrates the loss of ghrelin after Acanthisitta split from Eupasserines during the Palaeogene. While the receptor (GHS-R) is present even in Eupasserines, the ligand replacing ghrelin is still unknown. The phylogenetic tree was obtained from TimeTree (http://timetree.org/; last accessed 21 April 2023). The *x*-axis shows the age of the event in million years ago (Ma). Bird drawings were obtained from the Lynx Edicions’ *Handbook of the Birds of the World* with permission of Alada Gestió Empresarial S.L.

As a first step, we employed a tblastx search of the RefSeq protein sequences of ghrelin, MBOAT4, GHS-R and LEAP2 (from the chicken and the rifleman) against the complete avian National Center for Biotechnology Information (NCBI) Genomes database (WGS). We were not able to detect *ghrelin (GHRL*) in passerine birds (Passeriformes), except for the rifleman (*Acanthisitta chloris*). The split between Acanthisitti and Eupasseres is the oldest divergence within passerines, dated to around 50 Ma [15] or 56 Ma [16] (figure 1). We found *MBOAT4* to be present in several passerine bird genomes (13–18 species, depending on whether the chicken or the rifleman protein sequence was used). Blast hits ranged from 14% to 100% query coverage and from 28% to 78% identity. The chicken and rifleman *LEAP2* protein sequences matched partially to several passerine bird species. Ranging from 75% sequence overlap with 64.41% match similarity for the common starling (*Sturnus vulgaris*) to 39% sequence overlap with 93.10% match similarity for the zebra finch (*Taeniopygia guttat*a). *GHS-R* was present and complete in all investigated bird genomes. We further examined the presence of these genes specifically in the garden warbler (*S. borin*) genome (GCA_014839755.1), for which ghrelin had previously been shown to modify migratory behaviour [12]. We were only able to find *LEAP2* partially (37% coverage with a 96.9% similarity) and the complete *GHS-R* (100% coverage with a 91.5% similarity). Neither *ghrelin* nor *MBOAT4* showed any blast hits. Furthermore, a jackhmmer search did not identify *ghrelin* in any of the passerine genomes on the Ensembl database, other than the rifleman. However, the analysis identified *ghrelin* in several non-passerine bird genomes, thereby confirming our findings with BLAST.

We further investigated the presence of these genes in passerine birds using orthologue searches in the NCBI protein database, which resulted in similar findings. We only found annotations for ghrelin and MBOAT4 in passerines in the rifleman. Interestingly, while ghrelin showed a similar amino acid (aa) length in the rifleman (114 aa, XP_009080522.1) and the chicken (116 aa, XP_046782141.1), we found MBOAT4 to be much shorter in the rifleman (174 aa, XP_009071540.1) than in the chicken (424 aa, NP_001186218.2). However, several non-passerine birds showed similarly short MBOAT4 annotations, such as the red-throated loon (*Gavia stellata*) with 144 aa (XP_009819505.1) or the northern fulmar (*Fulmarus glacialis*) with 163 aa protein lengths (XP_009571461.1). We found LEAP2 orthologues for several passerine birds, ranging in predicted protein length from 75 aa to 119 aa, compared with 76 aa in the chicken (NP_001001606.1). *GHS-R* in the rifleman (344 aa, XP_009077327.1) showed a similar size to the chicken (347 aa, NP_989725.1). In general, the length of *GHS-R* seemed relatively constant with a length of 352 aa in most passerine birds, except for a few outliers such as the Tibetan ground-tit (*Pseudopodoces humilis*, 301 aa) or the Swainson’s thrush (*Catharus ustulatus*, 372 aa).

**Figure 2.**
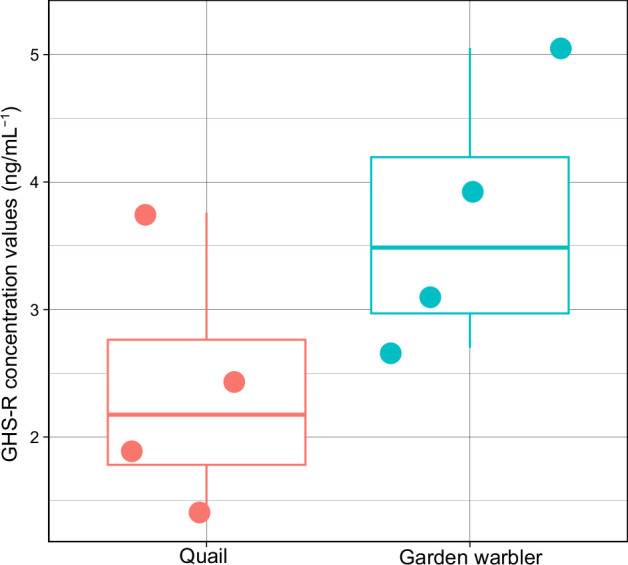
GHS-R abundance was measured using an enzyme immunoassay in plasma of the common quail and the garden warbler. Boxplot of the concentration values for GHS-R detected in plasma samples of the common quail and the garden warbler. Given the high homology of the GHS-R sequence within avian species, the chicken anti-GHS-R antibody was sensitive and cross-reacts with both species.

Although unlikely given the different techniques used to assemble the bird genomes on NCBI, we further investigated whether the general absence of *ghrelin* could be due to misassemblies in the genomes. To do so, we shotgun sequenced three garden warbler individuals with 30× genome-wide coverage. We then mapped the genomic reads to the chicken *ghrelin*, *MBOAT4*, *GHS-R* and *LEAP2* nucleotide sequences. We were not able to find a single-read mapping to either *ghrelin*, *MBOAT4* or *LEAP2*, but the entire coding sequence of *GHS-R* showed a coverage of 11×, 20× and 26× for the three individuals, respectively. Recently, analyses have indicated that genes with high GC content, such as the avian leptin, can be missing from genome assemblies due to sequencing biases [17]. Using deep RNA sequencing, several thought-to-be-missing genes in birds were recently discovered, including GC-rich genes (e.g. [18]). However, while *leptin* shows a GC content of about 70%, *ghrelin* has a GC content of about 38% and should thus not be affected by this sequencing bias. Furthermore, our blast search also included genomes generated with Oxford Nanopore Technologies’ long-read sequencing data (e.g. GCA_004360235.1), which show no GC-bias [19], and genomes generated with Pacific Biosciences (PacBio) long-read sequencing data (e.g. GCA_009819655.1), which show less GC-bias than Illumina-based sequencing methods (e.g. [20]); however, negative effects of GC content have been found in [21]. We further mapped RNAseq data (GenBank accessions: SRR23470326–SRR23470345) from [22], which was collected from garden warblers (*S. borin*) at migration stop-over sites, to *ghrelin* but found no reads mapping to it. Next, we employed a tblastn search of ghrelin, MBOAT4, GHS-R and LEAP2 against PacBio reads of the Eurasian blackcap (*Sylvia atricapilla*; GenBank accession: SRR25656515). We were only able to find reads blasting to GHS-R. In addition, we were able to identify and quantify the ghrelin receptor in the garden warbler plasma with an anti-GHS-R antibody. The detected receptor abundance could be identified in the range of approximately 1.5−3 ng ml^−1^ (figure 2).

*SEC13* and *IRAK2* are the flanking genes of ghrelin in the chicken genome. We found both genes present in the garden warbler genome, with the distance between exons of the two genes of a similar length in the chicken (approx. 13 kb) and the garden warbler (approx. 16 kb). Recently, *Seim et al.* [23] showed that the insertion of an ERVK retrotransposon in exon 0 led to the inactivation of *ghrelin* in falcons, which they hypothesize is an adaptation to a predatory lifestyle as it might increase food-seeking behaviour and feeding. We did not find any indication of a repeat insertion in the region between *SEC13* and *IRAK2* in the garden warbler. The presence of the two flanking genes on the same chromosomal region and the absence of a partial or inactivated ghrelin sequence suggest that ghrelin has been lost in Eupasserine birds, potentially via some sort of chromosomal rearrangement or pseudogenization.

### 2.2. Biochemical analysis of putative ghrelin homologues in garden warbler

To gather independent evidence for the identification of *ghrelin* in Eupasserines, we carried out immunoaffinity experiments combined with proteomic peptide analysis. We used plasma aliquots from the garden warbler and tissue from the Eurasian blackcap and the common quail to perform peptide profiling, along with two positive controls (plasma and tissue samples from the garden warbler spiked with chicken acylated ghrelin). We applied immunoaffinity chromatography with an anti-(rat) ghrelin rabbit antibody, which can specifically recognize acylated *n*-octanoyl ghrelin across different vertebrate species. The antibody is highly specific, able to react with the octanoyl modification but only at 0.3% or 20% to other modification forms such as *n*-hexanoyl or *n*-decanoyl chains [24,25]. Subsequently, we screened the peptide candidates using mass spectrometry, and the applied method was able to detect acylated ghrelin in the positive controls (table 1; electronic supplementary material, figure S1). However, de novo sequencing of the enriched peptide candidates by mass spectrometry did not result in a putative amino acid sequence with ghrelin-like motifs. The enriched peptides were in addition analysed by another mass spectrometry approach using matrix-assisted laser desorption ionization–time of flight mass spectrometry (MALDI TOF MS), observing no indicative [M+H]^+^ peptide ions within the range of *m/z* 3002.6 and *m/z* 3128.5. The fragmentation patterns could not confirm an octanoyl modification of serine or threonine with a mass difference of *m/z* 126. Thus, the nature of the ligand and its affinity towards the conserved GHS-R receptor at the molecular level remains to be unravelled in future studies.

**Table 1.** Ghrelin peptide analysis enriched by immuno-affinity chromatography and identified with mass spectrometry. Overview of the indicative fragments for ghrelin identification including peptide modification and sequence coverage. The LC-MS approach could specifically help to identify the acylated ghrelin standard in the spiked samples. In the garden warbler plasma and the Eurasian blackcap tissues, no peptide hormone with the unique octanoyl modification and S/T acylation could be identified. The * (asterisk) reflects the mass of the protonated peptide with and without the octanoyl (acyl-carrier peptide) chain modification with an m/z of 126.

sample	description	sequence	modification	mass [MH]+**DA**	peptides	sequence coverage (%)
positive control	appetite regulating hormone OS = *Gallus gallus* OX = 9031 Accession = Q7 T2V1	GS(+126)SLFLSPTYKNIQQQKDTRKPTARLH		3002.6/3128.6*	23	24
positive control	peptide OS = *Gallus gallus* OX = 9031 accession = Q7 T2V1	FLSPTYKNIQQQKDTRKPTARLH	2× deamidated	2772.4		
positive control	peptide OS = *Gallus gallus* OX = 9032 accession = Q7 T2V1	GSSLFLSPTYKNIQQQKDTRKPTARLH	2× deamidated	3003.5		
positive control	in solution tryptic peptide	NIQQQKDTR	2× deamidated	1132.5		
positive control	in solution tryptic peptide	NIQQQKDTRKPTAR	3× deamidated	1686.8		
garden warbler	not detected					
Eurasian blackcap	not detected					
quail	appetite regulating hormone OS = *Coturnix japonica* accession = AB244056	AGS(+126)SFLSPAYKNIQQQKNTRKPAARLHRR		3080.3/3206.3*	17	16

### 2.3. Evolutionary constraints on the growth hormone secretagogue receptor

Next, we investigated the conservation of *GHS-R* in passerine and non-passerine birds. The hypothesis would be that *GHS-R* shows little to no conservation in passerine birds if the pathway has been inactivated. We used the two sites selection models Fast, Unconstrained Bayesian AppRoximation (FUBAR; [26]) and single-likelihood ancestor counting (SLAC; [27]) to characterize patterns of selection in *GHS-R* using HyPhy [28]. Both FUBAR and SLAC indicated high proportions of sites under purifying selection in *GHS-R*. In all cases, the SLAC model inferred fewer sites under purifying selection than the FUBAR model. Both methods, however, indicated that passerine birds show considerably fewer sites under purifying selection (FUBAR: 76 out of 357 investigated sites, SLAC: 30 out of 357) than non-passerine birds (FUBAR: 223 out of 357, SLAC: 128 out of 357). To test if this differential amino acid constraint is indicative of a general relaxation of the *GHS-R* gene, we inferred whether the strength of natural selection has been significantly relaxed on the branch leading to the passerine birds, using the RELAX model in HyPhy. The absence of a significant relaxation signature (*K* = 1.14, *p* = 0.839, LR = 0.04) and the finding that injected ghrelin showed phenotypic effects in passerine birds (e.g. in [12,13] and [14]) indicate that the signalling pathway downstream of *ghrelin* is still functional and that a different agonist might be regulating this pathway. Furthermore, the adaptive branch-site random effects likelihood (aBSREL) and the branch-site unrestricted statistical test of episodic diversification (BUSTED) selection models did not find evidence of diversifying or purifying selection on the branch leading to the passerines. Preliminary work of our group suggests the existence of a putative ligand with sequence similarities to ghrelin-like motifs (data unpublished), which should correspond to the molecule detected in a femtomolar range by radioimmunoassay in previous studies in passerine birds [12,29]. To date, the nature of the ligand, i.e. its affinity to the receptor at the molecular level remains to be determined. However, another possible—but not likely—explanation is that the pathway is a relic, and while the receptor is still working, given the absence of the ligand, is on its way to be lost.

### 2.4. Evolutionary implications

These and previous findings suggest that in Eupasserine birds, the signalling pathway including and downstream of *GHS-R* still plays a role in food intake, and thereby maybe even migratory behaviour, but seems to be regulated by a hormone other than ghrelin. While *ghrelin* was lost in Eupasserines, *GSH-R* remained conserved during the evolution of vertebrates, including in Eupasserines, suggesting that ghrelin and its receptor evolved separately [30]. This is supported by the finding that to date there is no evidence for multiple *ghrelin* genes in any species [31].

Previous work has shown that birds diverged from mammals in the way they regulate food intake and fat deposition. The existence and role of leptin, the main adipostatic hormone in mammals, in avian species have been debated for decades, but it is now clear that birds do not use leptin as a feedback signal from the adipose tissue to regulate food intake (reviewed in [2]). Friedman-Einat & Seroussi [2] argue that in birds the ability to fly and migrate might reduce the threat of starvation, therefore, unlimited accumulation of fat reserves before migration could be more advantageous for survival than adipostatic control [2]. Our present study adds to this scenario that Eupasserine birds seem to have released themselves also from the second main system controlling food intake and fat metabolism in vertebrates, the ghrelin system. Most migratory passerines have a small body size, and therefore long-distance migratory species cannot carry sufficient energy stores to fuel the entire migratory journey like several shorebirds do [32]. Therefore, they rely on alternating rapid fuel deposition at stop-over sites and non-stop flights during which they exhaust most of their reserves. This peculiar, repeated alternation between very fat and lean conditions might have required to break away from conserved homeostatic control systems of vertebrates. Gene loss has been reported in several taxa as an adaptive mechanism that accompanies major transitions in physiology (reviewed in [33]), including adaptation to aquatic life in cetaceans such as diving ability or specific diets [34]. However, genes can also be lost by random chance and fixed by genetic drift or lost by neutral processes such as regressive evolution (reviewed in [35]). Given that migratory behaviour appears to be ancestral to the passerine lineage [36], the release from universal, conserved mechanisms controlling fat deposition and the consequent evolution of novel ways to regulate body mass might be behind the extreme success of this taxon, which alone comprises one-third of all avian species and 10% of all tetrapod diversity.

## 3. Material and methods

### 3.1. Genetic analyses

To investigate the presence of *ghrelin* and related genes in passerine bird genomes, we first downloaded the respective protein sequences of ghrelin (GenBank accession: NP_001001131.2), MBOAT4 (GenBank accession: NP_001186218.2), LEAP2 (GenBank accession: NP_001001606.1) and GHS-R (GenBank accession: NP_989725.1) for the chicken (*Gallus gallus*) from NCBI (ncbi.nlm.nih.gov/gene/). We further downloaded the same proteins for the rifleman (*Acanthisitta chloris*): ghrelin (GenBank accession: XP_009080522.1), MBOAT4 (GenBank accession: XP_009071540.1), LEAP2 (GenBank accession: XP_009082681.1) and GHS-R (GenBank accession: XP_009077327.1). We then blasted these sequences against the complete avian NCBI Genomes database (whole-genome shotgun contigs and taxid:8782; last accessed 20 March 2023) using tblastx for protein sequences (https://blast.ncbi.nlm.nih.gov/Blast.cgi) with default settings. We further downloaded the publicly available PacBio Sequel II read data of the Eurasian blackcap (*S. atricapilla*; GenBank accession: SRR25656515) and blasted the protein sequences of ghrelin, LEAP2, MBOAT4 and GHS-R against them using tblastn (https://blast.ncbi.nlm.nih.gov/Blast.cgi) with default settings. We further looked for these four genes in the NCBI RefSeq database (https://www.ncbi.nlm.nih.gov/refseq/). In addition, we used a jackhmmer search algorithm implemented in the EBI online hmmer server with default settings (https://www.ebi.ac.uk/Tools/hmmer/search/jackhmmer [37]) to investigate the presence of ghrelin in any of the passerine and non-passerine bird genomes on Ensembl (https://www.ensembl.org/index.html). We performed one round of searching to avoid overfitting.

Next, we generated genome libraries for three high-coverage garden warbler individuals after a modified protocol following Meyer & Kircher [38] (detailed laboratory methods can be found in electronic supplementary material, S1). Subsequently, we filtered the raw reads for quality, removed polymerase chain reaction (PCR) and optical duplicates and trimmed adapters using bbmap v. 38.87 [39]. To test whether the absence of *ghrelin* in the passerine genomes on NCBI could be due to assembly issues, we next mapped the genomic reads onto the chicken reference sequences of these genes and GHS-R (see above) using BWA 0.7.12-r1039 with the *mem* option [40] and sorted them using samtools 1.9 [41]. We then estimated the average coverage for GHS-R, as this was the only gene among those investigated in this work where reads were mapped to, using Qualimap v. 2.2.2-dev (option: *bamqc*; [42]). We checked for repeat insertions in the area between IRAK2 and SEC13, where ghrelin should be situated using RepeatMasker (https://repeatmasker.org/RepeatMasker/) in the garden warbler genome (GCA_014839755.1). We further tested whether we could find ghrelin expressed in RNAseq data from whole blood for *S. borin* available on NCBI (GenBank accessions: SRR23470326–SRR23470345; [22]). The species was chosen as (i) ghrelin administration showed an effect on migratory behaviour [12] and (ii) RNAseq data of individuals at migration stop-over sites are available (both from fat and lean individuals). We used Hisat2 [43] to map the reads against the ghrelin nucleotide sequence of the chicken and the rifleman (GenBank accession: XM_046926185.1 and NW_008653734.1).

To investigate patterns of molecular evolution of the *GHS-R* gene in passerine and non-passerine birds, we downloaded orthologue nucleotide sequences from NCBI (Aves, taxid:8782). These were then aligned using MAFFT [44]. We included only sequences that had a start codon at the beginning and a stop codon at the end of the sequence in the analyses. We further removed any sequences that showed internal stop codons. This resulted in 57 non-passerine and 30 passerine sequences for GHS-R (electronic supplementary material, table S1). We then carried out molecular evolution analyses using HyPhy [28] via the Datamonkey online server (http://www.datamonkey.org/ [45]); for passerines and non-passerines, respectively. We used the FUBAR (Fast, Unconstrained Bayesian AppRoximation [26], and the SLAC models [27]. We next investigated whether the reduced number of sites under purifying selection in passerine birds may result from selection relaxation using RELAX [46] via HyPhy [28] on the Datamonkey online server. We further applied branch-site selection models to investigate whether we find indications of diversifying or purifying selection on the branch leading to passerine birds (using the aBSREL [47] and BUSTED [48] models). We used *Crocodylus porosus* (GenBank accession: XM_019532518.1) and *Alligator mississippiensis* (GenBank accession: XM_006259227.3) as outgroups (electronic supplementary material, table S1).

### 3.2. GHS-R receptor quantification

The GHS-R concentration of plasma samples collected from two different bird species (the garden warbler and the common quail) was quantified using a commercially available enzyme-immunoassay kit for chicken ghrelin receptor (MyBioSource). The avian plasma samples were measured following the instructions of the manufacturer. Final concentrations were corrected for their respective dilution factors. We calculated the intra-assay coefficient of variation (CV) from concentrations of duplicate aliquots. Samples with an intra-assay CV above 20% were excluded from our analysis, and the mean CV of duplicates of all remaining samples was 5.9% ± 3.8% (mean ± s.d.). The detection limit reported by the manufacturer was 0.06 ng ml^−1^. Prior to analysing the individual samples, we successfully conducted a serial dilution of pooled warbler plasma to exclude any possibility of matrix effect. The GHS-R concentration for the two bird species was plotted using R (https://cran.r-project.org/) with the ggplot2 package (https://cran.r-project.org/web/packages/ggplot2/index.html).

### 3.3. Proteomic analyses

We extracted peptides from tissues (Eurasian blackcap and the common quail) according to [25,30] and from plasma samples (garden warbler) according to [49]. Briefly, liver and proventriculus samples were quickly frozen until further peptide purification. Next, we subjected the tissue samples to heat treatment and acidified the homogenate with 0.1 M HCl by mixing with a Polytron device. In parallel to the Eurasian blackcap tissue samples, commercially available chicken ghrelin (Phoenix Pharmaceuticals) was used for spiking proventriculus samples before and after immunoprecipitation. After a centrifugation step of 15 000*g* for 10 min the supernatant was subjected to solid phase extraction (SPE) with hydrophilic lipophilic balance (HLB) material. We then eluted the enriched peptides with 60% acetonitrile and 0.1% trifluoroacetic acid. The peptides were subsequently subjected to an immune affinity purification step using Sepharose Immunolink beads (Pierce) coated with anti-*ghrelin* antibody made in rats pre-incubated with the enriched peptide ligands at 4°C under constant rotation. We mixed the ligand antibody mixture with the Protein A/G coated beads for another hour, and after serial washing steps, the ligand was eluted and after lyophilization repurified with solid phase extraction by SPE (C18 material-Supelco). The peptides (diluted in 50 mM TRIS to a final concentration of 20 ng µl^−1^ for the internal chicken ghrelin standard) were subject to a reducing and alkylation step using 50 mM dithiothreitol (DTT) for 30 min at 37°C and 200 mM iodoacetamide prior to liquid chromatography separation and mass spectrometry analysis. Furthermore, using the PEAKs software, peptide candidates were sequenced de novo. Briefly, we separated the peptides on a nano-HPLC Ultimate 3000 RSLC system (Dionex). Sample pre-concentration and desalting were accomplished with a 5 mm Acclaim PepMap μ-Precolumn (300 µm inner diameter, 5 µm particle size and 100 Å pore size) (Dionex). For sample loading and desalting, we used 2% acetonitrile (ACN) in ultra-pure H_2_O with 0.05% trifluoroacetic acid (TFA) as a mobile phase with a flow rate of 5 µl min^−1^. Separation of peptides was performed on a 25 cm Acclaim PepMap C18 column (75 µm inner diameter, 2 µm particle size and 100 Å pore size) with a flow rate of 300 nl min^−1^. The gradient started with 4% B (80% ACN with 0.08% formic acid) for 7 min, increased to 31% in 30 min and to 44% in an additional 5 min. This was followed by a washing step with 95% B. Mobile Phase A consisted of ultra-pure H_2_O with 0.1% formic acid.

For the liquid chromatography-mass spectrometric (LC-MS) analysis, the liquid chromatography was directly coupled to a high-resolution Q Exactive HF Orbitrap mass spectrometer. We performed MS full scans in the ultrahigh-field Orbitrap mass analyser in ranges *m/z* 350−2000 with a resolution of 60 000, the maximum injection time (MIT) was 50 ms and the automatic gain control (AGC) was set to 3 × 10^6^. We subjected the top 10 intense ions to Orbitrap for further fragmentation via high-energy collision dissociation activation over a mass range between *m/z* 200 and 2000 at a resolution of 15 000 with the intensity threshold at 4 × 10^3^. Ions with charge states +1, +7, +8 and greater than +8 were excluded. Normalized collision energy was set at 28. For each scan, the AGC was set at 5 × 10^4^ and the MIT was 50 ms. We used dynamic exclusion of precursor ion masses over a time window of 30 s to suppress repeated peak fragmentation.

Finally, we carried out a database search using the Proteome Discoverer Software 2.4.1.15 (Thermo Fisher Scientific) and PEAKS with the respective taxonomy settings for all avian species analysed and the contamination databases provided from the Uniprot database (e.g. https://www.uniprot.org/uniprotkb: chicken_tx9031, garden warbler tx 73 324 and quail tx 93 934 and the https://www.thegpm.org for the contaminants) For the database search, a maximum of two sites missed cleavages for trypsin digestion products was entered with a precursor mass tolerance of 10 ppm and 0.02 for the fragment ions. The dynamic modifications for peptide assignment were oxidation +15.995 Da (M) and deamidation +0.984 Da (N, Q). Targeted false discovery rate (FDR), fixed and relaxed were set as 0.01 and 0.05 for the PSM validator software (Thermo Fisher Scientific). The plasma-enriched peptides were analysed with an additional ionization technique provided by an Autoflex Speed MALDI TOF MS (Bruker) machine. The peptides were spotted on a stainless-steel target plate and measured in positive ion mode; the acquired data were analysed using the manufacturer’s software (Flexanalysis, Bruker).

## Data Availability

Genomic reads of the three garden warbler (*Sylvia borin*) samples can be found on NCBI BioProject PRJNA975133. Supplementary material is available online [50].
